# Characteristics and Outcomes of *Acinetobacter baumannii* Infections in Patients with HIV: A Matched Case-Control Study

**DOI:** 10.1038/s41598-018-33753-9

**Published:** 2018-10-23

**Authors:** Junyang Yang, Qi Tang, Tangkai Qi, Jun Chen, Yongjia Ji, Yang Tang, Zhenyan Wang, Wei Song, Jingna Xun, Li Liu, Yinzhong Shen, Renfang Zhang, Hongzhou Lu

**Affiliations:** 1 Department of Infectious Diseases, Shanghai Public Health Clinical Center, Fudan University, Shanghai, China; 2 Wenzhou Medical University, Wenzhou, Zhejiang China; 3 Scientific Research Center, Shanghai Public Health Clinical Center, Fudan University, Shanghai, China; 4 Department of Infectious Disease, Huashan Hospital Affiliated to Fudan University, Shanghai, China

## Abstract

*Acinetobacter baumannii* (AB) infection is an increasing global threaten to hospitalized patients, especially those with impaired immune function. Still, few studies addressed the disease burdens and outcomes of AB infection in HIV patients. We aimed to describe characteristics and outcomes of AB infections in patients with HIV, measure the impact of AB infection on 28-day mortality in HIV patients, as well as assess the predictors of 28-day survival among HIV patients with AB pneumonia. A retrospective study with HIV/AB co-infected patients was conducted at Shanghai Public Health Clinical Center (SPHCC), China. Patients with AB pneumonia were further analyzed for predictors of mortality, as well as an additional 1:1 case-control study to determine the fatality of AB pneumonia compared with pneumonia of other pathogens. We found the incidence of AB infection was 17.4 cases per 100 person-years among all hospitalized HIV patients. Hospital mortality rate was 37.5% (21/56). There was a higher 28-day mortality rate in HIV patients with pneumonia due to AB than other pathogens (34% vs 16%, *P* = 0.03). APACHE II score was independently associated with 28-day survival by multivariate logistic regression (*P* = 0.031). Our findings indicate that AB infection is incident and can be fatal in HIV seropositive population. AB infection is an independent risk factor of mortality in patients with HIV and pneumonia. A lower APACHE II score on admission predicts a higher 28-day survival rate among HIV/AB co-infected patients.

## Introduction

Bacteria of the species *Acinetobacter baumannii* (AB) are notorious for their potential to spread epidemically in hospitals^1,2^ and for their resistance to multiple antibiotics as has been observed worldwides^3,4^. Twelve out of 27 countries reported a 50% or higher prevalence of carbapenem resistance among AB isolates^5,6^.

AB may cause various types of infections, such as pneumonia, urinary tract infections, bacteremia, and wound infections. Reports showed that the mortality rate attributable to AB infection ranged from 7.8% to 23% outside intensive care units (ICUs) and from 10% to 43% in ICUs^7^. Resistance to carbapenems is a significant predictor of mortality among AB patients (adjusted odds ratio: 2.49; 95% CI: 1.61–3.84)^8^.

Patients with immune suppression, serious underlying diseases, as well as subject to invasive procedures and/or broad-spectrum antibiotics, are especially vulnerable to AB infection. Patients with human immunodeficiency virus (HIV) infection, if not treated in time, will finally develop to acquired immunodeficiency syndrome (AIDS) with life-threatening opportunistic infections and malignancies^9–11^.

HIV seropositive individuals are also at risk of bacterial infections. Even in the combined antiretroviral therapy (cART) era, HIV seropositive individuals are at a 6–8 fold higher risk of pneumonia than HIV seronegative individuals^12^. Furthermore, an analysis of the EuroSIDA cohort reported a higher incidence of severe bacterial non-AIDS infections compared with other non-AIDS defining events such as non-AIDS cancers, cardiovascular events and chronic kidney dysfunction^13^. Thus, bacterial infection is an important complication in HIV seropositive individuals. Therefore, HIV seropositive individuals may be susceptible to AB infection.

Still, little is known about AB infection in this population. Limited data showed that AB infection or colonization was more fatal in AIDS patients compared with HIV seronegative patients^14^. It is not clear whether AB infection is more fatal than other bacterial infections among HIV seropositive individuals. Neither do we know the determinants of mortality of AB infections in this population.

Given the increasing global burden of AB infections^15^, and limited literature available about the disease among HIV seropositive population, we conducted this case–control study to discuss the characteristics and outcomes of AB infections in patients with HIV.

## Materials and Methods

### Study designing and setting

A retrospective observational study was done at Shanghai Public Health Clinical Center (SPHCC) affiliated to Fudan University. The SPHCC is a designated medical institution that provides outpatient and inpatient care for people living with HIV (PLWH) in Shanghai as well as East China, including more than 8000 admissions of HIV patients over the last decade. This study was approved by the institutional review board of SPHCC with a waiver of informed consent for the collection and analysis of retrospective data.

HIV seropositive individuals admitted to the center between January 2010 and December 2016 was screened for AB infection by the electronic medical system. Outcomes are defined as hospital mortality and 28-day mortality. Among them, patients with pneumonia due to AB were selected to analyze potential risk factors of mortality, such as age, gender, length of hospitalization, the need for mechanical ventilation, AB resistance pattern, ART naive or experienced, CD4^+^ T lymphocyte count, CD4/CD8 ratio and APACHE II score. A retrospective 1:1 case-control analysis was done to measure the fatality rate of pneumonia due to AB or other bacteria in HIV positive patients. The control group was comparable with the case group in factors above except for AB resistance pattern, which was unavailable in the control group. Each pair of case and control were matched in admission date (within 1 year).

### Patients

*Inclusion Criteria*: *i*) A positive result of anti-HIV by ELISA and Western Blot; *ii*) positive AB cultures in any a biological specimen *iii*) clinical manifestation with or without radiological findings in adherence with AB infection of the culture site. For AB pneumonia, additionally require: *iv*) AB pure or predominant culture in at least 2 eligible sputum.

*Exclusion Criteria*: *i*) Growth of AB and other bacteria together in one culture, with another bacterium as the predominant strain; *ii*) AB colonization without relevant clinical manifestation; *iii*) patients who did not receive antimicrobial therapy.

Demographic data of patients, including age, sex, dates of admission and discharge, was collected from the electronic medical record system. So was the routine blood test, biochemistry data, antibiotic treatment, usage of tigecycline, CD4^+^ T lymphocyte count, CD4/CD8 ratio, microbiological culture, antibiotic resistance and other potential predictors. APACHE-II score was retrospectively calculated making use of data from the medical record system, including vital signs, fraction of inspired oxygen, and consciousness on admission, as well as age and need for surgery. Immune dysfunction was default for each a patient. Patients was recorded until death in hospital or discharge. Those discharged within 28 days after admission was followed by telephone call to determine the outcome.

### Microbiology

Blood specimen were collected from at least 2 sites and then incubated in BacT/ALERT3D® system (bioMérieux, Marcy l’Etoile, France) for 7 days. Sputum, urine, stool, or other specimens were cultured on blood plate, bloodthirsty chocolate plate, and/or China blue agar plate, respectively. The identification of species was made by standard phenotypic tests including API 20 NE (bioMe’rieux, Marcy l’Etoile, France). Compared with PCR assays, phenotypic methods have limitation in distinguish AB from other species of the AB complex, such as A. pittii, A. nosocomialis and A. seifertii^16^. Thus, AB in this article refer to the AB complex. Antimicrobial susceptibility testing was performed manually with disk diffusion method. All the procedures were in adherence to the NCCLS standards.

We classified the AB isolates into multidrug-resistant (MDR), extensively drug-resistant (XDR), pandrug-resistant (PDR) and non drug-resistant (NDR). MDR was defined as resistant to all penicillins, cephalosporins, fluoroquinolones, and aminoglycosides. XDR was defined as resistant to the three classes listed above and resistant to carbapenems. PDR was defined as resistant to polymyxins and tigecycline in addition to XDR. NDR hereby referred to susceptible to at least 2 classes out of penicillins, cephalosporins, fluoroquinolones, aminoglycosides and carbapenems.

### Statistical analysis

Demographic data were presented as means with standard deviation, medians with interquartile ranges or percentages, respectively. The *t* test or Mann-Whitney U test was used to compare the continuous data, and Pearson’s χ^2^ test or Fisher’s exact test was used to compare the categorical data in the two groups. Kaplan-Meier survival analysis was employed to compare survival between the two groups. Logistic regression was performed to explore the risk factors for mortality at 28 days in patients with AB pneumonia. All the statistics were done with SPSS (version 19.0), and *P* < 0.05 was considered statistically significant for all tests.

## Results

### Characteristics and outcomes of AB infections in patients with HIV

During the period from January 2010 to December 2016, 6935 HIV-infected patients were admitted to the SPHCC. Among those patients, 56 (0.81%) patients had AB infection during their hospital stay. The incidence rate of AB infection among hospitalized HIV patients was 4.78 cases per 10,000 person-days (56/117259), or 17.4 cases per 100 person-years. The prevalence rate of HIV/AB co-infection was 0.72% (4/555), 0.71% (5/701), 0.95% (8/845), 0.21% (2/935), 0.96% (11/1148), 0.45% (6/1312), 1.4% (20/1439) during individual year of the study period, successively. We did not find a difference in the prevalence rate between years.

Characteristics and outcomes of HIV/AB co-infected patients were described in Table 1. Of 56 HIV/AB co-infected patients, the mean age was 42.2 ± 13.5 years, and 52 (92.9%) patients were male. The median CD4 + T lymphocyte countwas 19.0 (6.0–59.5) cells/μL. The median APACHE II score was 17.5 (14.3–22.0). 24 (42.9%) patients required mechanical ventilation during hospital stay.

**Table 1:** Characteristics and outcomes of patients with HIV and *A. baumannii* infections.

Characteristics	*n* = 56
Age	42.2 (13.5)
Male	52 (92.9%)
Length of stay	40.3 (28.3)
**Site of infection**	
Respiratory	50 (89.3%)
Blood	2 (3.6%)
Urinary tract	2 (3.6%)
Intestinal tract	1 (1.8%)
Needle biopsies from abscess	1 (1.8%)
Mechanical ventilation	24 (42.9%)
**Resistance pattern**	
MDR	8 (14.3%)
XDR	16 (28.6%)
PXR	17 (30.4%)
NDR	15 (26.8%)
ART experienced	14 (25.0%)
Therapy including Tigecycline	16 (28.6%)
CD4 + T lymphocyte count	19.0 (6.0–59.5)
CD4/ CD8 ratio	0.08 (0.02–0.14)
APACHE II score on admission	17.5 (14.3–22.0)
**Outcomes**	
Prognosis	
Survival	35 (62.5%)
Hospital mortality	21 (37.5%)

ART: antiretroviral therapy; MDR: multidrug resistant; NDR: non-drug resistant; PDR: pan-drug resistant; XDR: extensively drug-resistant.

The AB positive cultures were primarily obtained from the respiratory tract (89.3%). The rest were from blood (3.6%), urinary tract (3.6%), intestinal tract (1.8%) and needle biopsies for abscess (1.8%), respectively.

Fourteen (25.0%) patients had received ART before admission, and the rest of them started ART during hospitalization.

Overall hospital mortality rate was 37.5% (21/56), and 28-day mortality rate was 35.6% (20/56). Hospital mortality rate among patients with MDR-AB, XDR-AB, PDR-AB and NDR-AB was 25% (2/8), 37.5% (6/16), 41.2% (7/17) and 40% (6/15), respectively.

Each patient was given empirical antibacterial therapy after collecting specimens for culture, then antibacterial agents would be modified according to the antibiotic susceptibility test and clinical efficacy. Antibiotics ever used included: amikacin, clindamycin, osfomycin, norvancomycin, sulfamethoxazole–trimethoprim, cefperazone-Sulbactam, imipenem-Cilastatin, azithromycin, meropenem, cefaclor, ceftriaxone, Tigecycline, moxifloxacin, levofloxacin, piperacillin-tazobactam, isepamicin, biapenem and so on. Sixteen (28.6%) of them were given a therapy including tigecycline.

### Mortality and survival at 28 days in the case and control groups

Given the predominant infection site was the lung, 50 patients with AB pneumonia were selected for the matched case–control study to control the bias. Control group consisted of 50 patients with pneumonia due to other nosocomial bacterial, including *Klebsiella pneumonia* (*n* = 13), *Coagulase negative Staphylococcus* (*n* = 9), *Pseudomonas aeruginosa* (*n* = 9), *Enterococcus Escherichia coli* (*n* = 5), *Staphylococcus aureus* (*n* = 5), *Enterobacter cloacae* (*n* = 3), *Enterococcus faecalis* (*n* = 2), *Proteus mirabilis* (*n* = 1), *Indolonus indolens* (*n* = 1), *Stenotrophomonas maltophilia* (*n* = 1) or *Burkholderia cepacia* (*n* = 1), respectively. The case and control group are comparable in age, gender, length of hospital stay, mechanical ventilation, history of ART, CD4^+^ T lymphocyte count and APACHE II score, as presented in Table 2.

**Table 2:** Characteristics and outcomes of the case and control group.

Variable	Case group (*n* = 50)	Control group (*n* = 50)	*P* value
	HIV Patients with AB pneumonia	HIV Patients with pneumonia other than AB	
**Characteristics**			
Age	41.9 (13.8)	44.3 (13.3)	0.30
Male	46 (92%)	47 (94%)	0.50
Length of stay	37.4 (25.3)	35.1 (23.9)	0.62
Mechanical ventilation	24 (48%)	21 (42.0%)	0.34
ART	10 (20%)	19 (38%)	0.08
CD4^+^ T lymphocyte count	18.5 (5.0–54.3)	27.5 (8.0–87.0)	0.07
APACHE II score	18.5 (15.0–23.3)	15.0 (13.8–19.3)	0.08
**Outcomes**			
Mortality, n (%)	17 (34%)	8 (16%)	0.03

ART: antiretroviral therapy.

**Figure 1:**
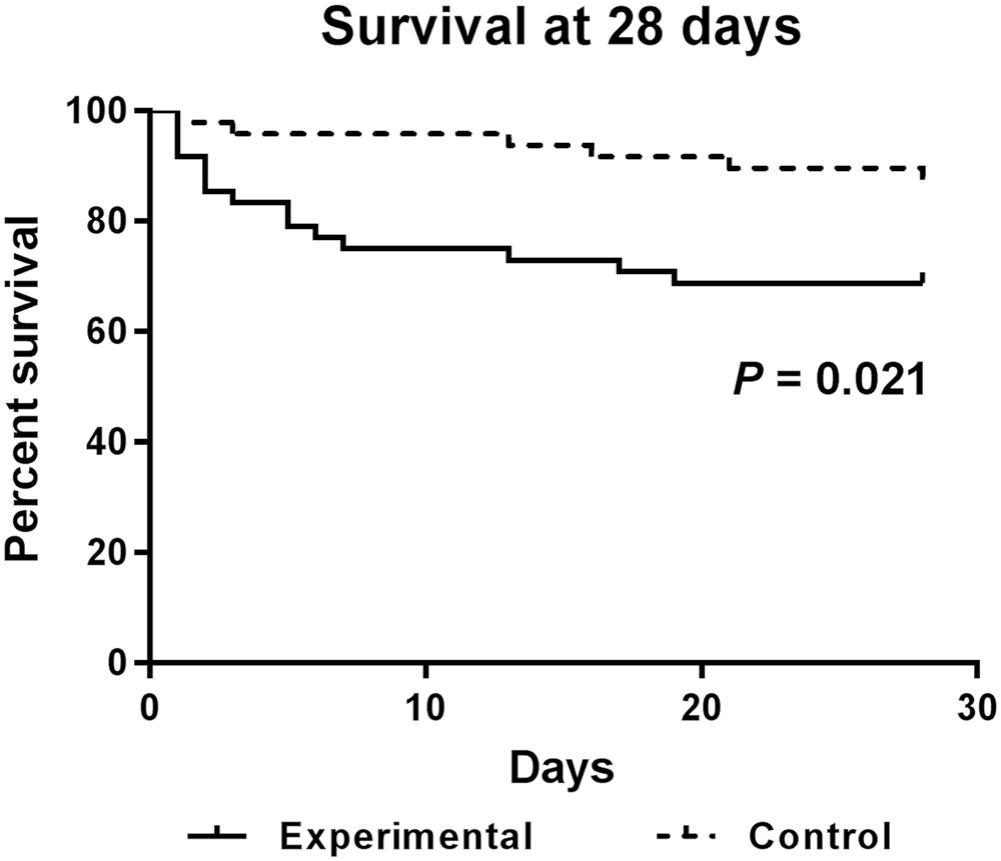
Survival of case and control group at 28 days of hospitalization. The graphs compare the survival status between the case and control groups. The results indicated that the control group achieved higher survival rates comparing with the case group with a significant difference (*P* = 0.021).

The mortality rate of the case group was significantly higher than the control group (34% vs 16%, *P* = *0.03*). We also conducted a survival curve at 28 days for the two groups (Fig. 1). The 28-day survival rate for the case group and the control group was 66% (33/50) and 84% (42/50), respectively. The control group achieved a significantly higher survival rate compared with the case group (*P* = 0.021).

### Predictors of 28 day survival among patients with AB infections

Among the 50 HIV patients with AB pneumonia, the predictors of 28 day survival were given special attention. Logistic regressions of univariate analysis and multivariate analysis were performed to sort out these risk factors (Table 3).

**Table 3:** Predictors of 28 day survival among patients with *Acinetobacter baumannii* infections.

Clinical Characteristic	28 Day Survival		Univariate *P*-value	OR (95%CI)	Multivariate *P*-value	OR (95%CI)
	Alive (n = 33)	Dead (n = 17)				
Age	40.9 (11.6)	43.6 (17.5)	0.51	0.99 (0.94–1.03)	—	—
Male	29 (87.9%)	17 (100%)	0.18	0.63 (0.51–0.79)	—	—
Length of stay	41.3 (22.1)	29.9 (29.9)	0.14	1.02 (0.99–1.05)	—	—
Mechanical ventilation	11 (33.3%)	13 (76.5%)	0.004	6.50 (1.71–24.68)	0.59	1.62 (0.27–9.64)
Resistance pattern			0.98	0.99 (0.56–1.77)	—	—
MDR	5 (15.2%)	2 (11.8%)				
XDR	8 (24.2%)	4 (23.5%)				
PXR	10 (30.3%)	7 (41.2%)				
NDR	10 (30.3%)	4 (23.5%)				
CD4^+^ T lymphocyte count	19.0 (6.0–54.5)	8.0 (4.5–62.0)	0.57	0.99 (0.98–1.01)	—	—
CD4/ CD8 ratio	0.08 (0.02–0.13)	0.05 (0.02–0.11)	0.68	5.14 (0.02–12.10)	—	—
APACHE II score	16.0 (12.0–21.5)	22.0 (19.0–26.0)	0.002	1.26 (1.09–1.45)	0.031	1.22 (1.02–1.45)
ART	7 (21.2%)	3 (17.6%)	0.77	0.80 (0.18–4.57)	—	—
Tigecycline	11 (33.3%)	4 (23.5%)	0.48	0.62 (0.16–2.34)	—	—

ART: antiretroviral therapy; MDR: multidrug resistant; NDR: non-drug resistant; OR: odds ratio; PDR: pan-drug resistant; XDR: extensively drug-resistant.

Thirty-four percent (17/50) of the patients died within 28 days after admission. The potential variables included age, gender, duration of hospitalization, mechanical ventilation, AB resistance pattern, CD4^+^T lymphocyte count, CD4/CD8 ratio, APACHE II score, ART experienced or naiive, as well as therapy including tigecycline.

By univariate logistic regression, survival rate at 28 days was significantly lower in patients using mechanical ventilation (*P* = 0.004), however, there was no significant difference by multivariate logistic regression (*P* = 0.59). We did not find an association between 28-day survival and factors such as CD4^+^ T lymphocyte count, usage of tigecycline, history of ART.

Overall, the results indicated that the APACHE II score was independently associated with 28 day survival by multivariate logistic regression [*P* = 0.031, OR = 0.82 (0.69–0.98)]. Twenty-eight day survival was significantly higher for patients who had lower APACHE II score compared to those with higher scores.

## Discussion

AB can cause a broad range of severe nosocomial infections, including skin and soft tissue infections, wound infections, urinary tract infections and secondary meningitis^17^. Still, there is little knowledge about the disease burden, characteristics and outcomes of AB infections in patients with HIV.

The incidence rate of AB infection varies in different settings. Van den Broek, *et al*. reported that the incidences of *Acinetobacter* isolates ranged from 1.7 to 3.7 per 10,000 patient-days in a Dutch university hospital^18^. A study in South Africa reported the incidence of *A.baumannii* colonisation/infection in ICUs was 15 per 100 person-years^14^. A meta-regression concluded that the worldwide incidence of *Acinetobacter* associated ventilator associated pneumonia ranged from 12–88 per 10,000 mechanical ventilation days^19^.

In this study among HIV seropositive inpatients, we reported an incidence rate of 4.78 cases per 10,000 person-days, in other words, 17.4 per 100 person years. Although the incidence rate is not so high as in mechanical ventilated population, it is higher than the number in general patients and comparable with that in ICU. This indicates that immune dysfunction in HIV seropositive patients may facilitate the occurrence of AB infection. Given the huge amount of HIV/AIDS patients over the world, their co-infection due to AB may be an alarming public health issue. This highlights the urgency of infection prevention and control against AB, especially in medical facilities serving for patients including those infected with HIV^14^.

A systematic review of data up to May 2013 concluded a 33% (850/2545) mortality rate among AB infections in 16 observational studies^8^. In our study, hospital mortality rate and 28-day mortality rate for 56 patients with HIV/AB co-infection was 37.5% and 35.6%, respectively. The data is high, and comparable with previous study as well. In 1:1 case-control study, after controlling potential risk factor factors, we found a higher 28-days mortality rate in HIV patients with pneumonia due to AB than other nosocomial bacteria (34% vs 16%, *P* = 0.03). This calls for further insight into factors influencing the outcome of AB infection in HIV patients.

Studies have shown that the immunosuppression can lead to an increase in 30-day mortality of AB infection^7^. We did not find an association between CD4^+^ T lymphocyte count and mortality. This may be partially restricted by low level of CD4^+^ T lymphocyte counts in a majority of the subjects. Out of a bundle of potential risk factors, APACHE-II score was positively associated with mortality rate by multivariate analysis. This give prominence to intensive care in treating patients with HIV and AB infection.

Among 27 European countries, 12 reported that over 50% of AB isolates were resistant to carbapenems^5^. WHO claimed that CRAB is one of potential threatens to the stability of the health care system in both the short and long term^15^. In our study, XDR-AB and PDR-AB was isolated in 16 and 17 patients, respectively, implying that no less than 58.9% of AB strains were resistant to carbapenems. This favors the coverage of carbapenem resistant strains in empirical treatment for HIV patients at risk of AB infection.

*In vivo* study and murine model has shown satisfactory activity of tigecycline against AB^20,21^. However, in a guideline published on 2016, IDSA recommend against the use of tigecycline in patients with HAP/VAP caused by Acinetobacter species^22^. The recommendation was mainly based on 2 matched cohort studies, showing that tigecycline was non-superior than imipenem and inferior to colistin, respectively^23,24^. In our study, tigecycline therapy failed to reduce the mortality of HIV/AB co-infection. We should be cautious when interpreting the data, given the relative small sample size and huge variation in drug-resistance of AB among different settings. Decision of tigecycline usage in HIV/AB co-infections should be cautious, considering drug-resistance data, individual factors, as well as drug availability and affordability.

There are certain limitations in our study. First, the sample size is not so large compared to studies in the general population. However, this is the largest to date in HIV/AB co-infection. Second, data was collected retrospectively and bias were inevitable. Future prospective studies may better control the bias. Third, colistin and polymyxin B was not available in China during the study period, thus we failed to provide data about these drugs. Future usage of polymyxin B should follow literatures such as the IDSA guidelines^22^. Also, the commercial phenotypic systems used in this study could not differentiate *A. baumannii* from several closely related species, such as from several close strains of Acinetobacter such as *A. pittii*, *A. nosocomialis* and *A. seifertii*^19,25^. Thus AB in this article actually refers to the *Acinetobacter baumannii* complex. The hospital has recently applied MALDI-TOF MS assay to better identify the species. Future study should present data from state-of-the-art molecular techniques.

In conclusion, the study reveals the burden and fatality of AB infection among HIV seropositive population, highlighting the need for infection prevention and control against the bacterial. The data may benefit therapeutic decision making for HIV/AB co-infection, including empirical treatment and the usage of tigecycline. Rather than indicators of immune suppression, APACHE-II score was found to be associated with mortality, giving prominence to intensive care when managing patients with HIV and AB infection. We expect larger multi-center prospective study covering a wider range of sub-populations and employing more state-of-the-art molecular techniques in the future.
